# Supplementary material for: Influence of Intrauterine Inflammation, Delivery, and Postnatal Feeding on the Temporal Changes of Serum Alpha 1 Acid Glycoprotein Levels in Extremely-Low-Birth-Weight Infants
Source: Nutrients. 2022 Dec 4;14(23):5162. doi: 10.3390/nu14235162 (PMC9736676; doi:10.3390/nu14235162)
Supplement: Supplementary file 1 [file nutrients-14-05162-s001.zip › nutrients-2026904-supplementary.pdf]

Online Supplementary Information

Table S1. Crude relationships between clinical variables

		1	2	3	4	5	6	7	8	9	10	11	12	13	14	15	16	17	18	19	20
**1**	Antenatal steroid	1	-0.029	-0.031	.206**	-0.031	-0.011	-0.057	.222**	.140*	-0.053	.203**	.144*	0.001	-0.075	0	0.001	0.085	0.102	.181**	-0.061
**2**	PROM	-0.029	1	-.460**	.136**	-0.001	.307**	-0.113	-.524**	-.229**	.428**	-.156*	-0.191*	.175**	-0.021	-0.044	.316**	-0.024	-0.027	-0.006	0.030
**3**	HDP	-0.031	-.460**	1	.157**	-.241**	-.206**	.206**	.438**	0.088	-.467**	.183**	.203**	.202**	.121*	-.120*	-.281*	0.004	0.058	0.059	0.046
**4**	Maternal antibiotics	.206**	.136**	.157**	1	0.089	0.041	0.019	0.096	-0.070	-.204**	-0.022	-0.064	.486**	0.029	0.045	0.104	0.047	-0.072	-0.015	0.058
**5**	Chorioamnionitis	-0.031	-0.001	-.241**	0.089	1	.290**	-.292**	-.135*	0.088	.206**	-0.016	-0.035	-.295**	-0.045	-0.006	-0.006	-0.055	.126*	.127*	.226**
**6**	Funisitis	-0.011	.307**	-.206**	0.041	.290**	1	-.174**	-.384**	-0.111	.376**	-.164**	-.201**	-.370**	-0.061	-0.042	.234**	-.216**	.157*	.205**	.159**
**7**	Male sex	-0.057	-0.113	.206**	0.019	-.292**	-.174**	1	.215**	-0.058	-.151*	-.143*	-0.079	.127*	-0.079	-.130*	-0.093	0.034	-0.007	-0.068	-.133*
**8**	Gestational age	.222**	-.524**	.438**	0.096	-.135*	-.384**	.215**	1	.524**	-.689**	.420**	.453**	.128*	-0.055	-0.077	-.442**	-0.081	0.112	-0.017	-.134*
**9**	Birth weight	.140*	-.229**	0.088	-0.070	0.088	-0.111	-0.058	.524**	1	.151*	.480**	.528**	-0.100	-.187**	-.146*	-.336**	-0.096	174**	-0.053	-0.008
**10**	z-score of birth weight	-0.053	.428**	-.467**	-.204**	.206**	.376**	-.151*	-.689**	.151*	1	-.159**	-.151*	-.319**	-0.114	-0.037	.285*	0.010	0.066	-0.041	.148*
**11**	Apgar score (1 min)	.203**	-.156*	.183**	-0.022	-0.016	-.164**	-.143*	.420**	.480**	-.159**	1	.780**	0.083	0.110	-.146*	-.356**	-.130*	.152*	.148*	-0.103
**12**	Apgar score (5 min)	.144*	-0.191*	.203**	-0.064	-0.035	-.201**	-0.079	.453**	.528**	-.151*	.780**	1	0.096	0.110	-0.100	-.416**	-.226**	0.104	0.107	-0.027
**13**	Caesarean delivery	0.001	.175**	.202**	.486**	-.295**	-.370**	.127*	.128*	-0.100	-.319**	0.083	0.096	1	-0.061	0.040	-0.113	0.101	-.206**	-.190**	-.133*
**14**	Septicemia	-0.075	-0.021	.121*	0.029	-0.045	-0.061	-0.079	-0.055	-.187**	-0.114	0.110	0.110	-0.061	1	-0.023	-0.053	-0.063	0.001	0.044	.203**
**15**	Intestinal perforation	0	-0.044	-.120*	0.045	-0.006	-0.042	-.130*	-0.077	-.146*	-0.037	-.146*	-0.100	0.040	-0.023	1	.145*	-0.046	-.132*	-0.087	0.055
**16**	PDA requiring surgery	0.001	.316**	-.281*	0.104	-0.006	.234**	-0.093	-.442**	-.336**	.285*	-.356**	-.416**	-0.113	-0.053	.145*	1	.169**	-0.070	0.039	.221**
**17**	Grade III/IV IVH	0.085	-0.024	0.004	0.047	-0.055	-.216**	0.034	-0.081	-0.096	0.010	-.130*	-.226**	0.101	-0.063	-0.046	.169**	1	-.151*	-.183**	-0.014
**18**	Enteral nutrition	0.102	-0.027	0.058	-0.072	.126*	.157*	-0.007	0.112	.174**	0.066	.152*	0.104	-.206**	0.001	-.132*	-0.070	-.151*	1	.553**	-0.013
**19**	Probiotics	.181**	-0.006	0.059	-0.015	.127*	.205**	-0.068	-0.017	-0.053	-0.041	.148*	0.107	-.190**	0.044	-0.087	0.039	-.183**	.553**	1	0.047
**20**	C-reactive protein	-0.061	0.030	0.046	0.058	.226**	.159**	-.133*	-.134*	-0.008	.148*	-0.103	-0.027	-.133*	.203**	0.055	.221**	-0.014	-0.013	0.047	1

Regression coefficients are from the Spearman rank-ordinal test.

Abbreviations: HDP, hypertensive disorders of pregnancy. IVH, intraventricular hemorrhage. PDA, patent ductus arteriosus. PROM, premature rupture of membranes.

P-values < 0.05* or < 0.01** without correction for multiple comparisons.

Figure S1. Relationships between clinical variables and alpha 1 acid glycoprotein levels at early and late postnatal ages

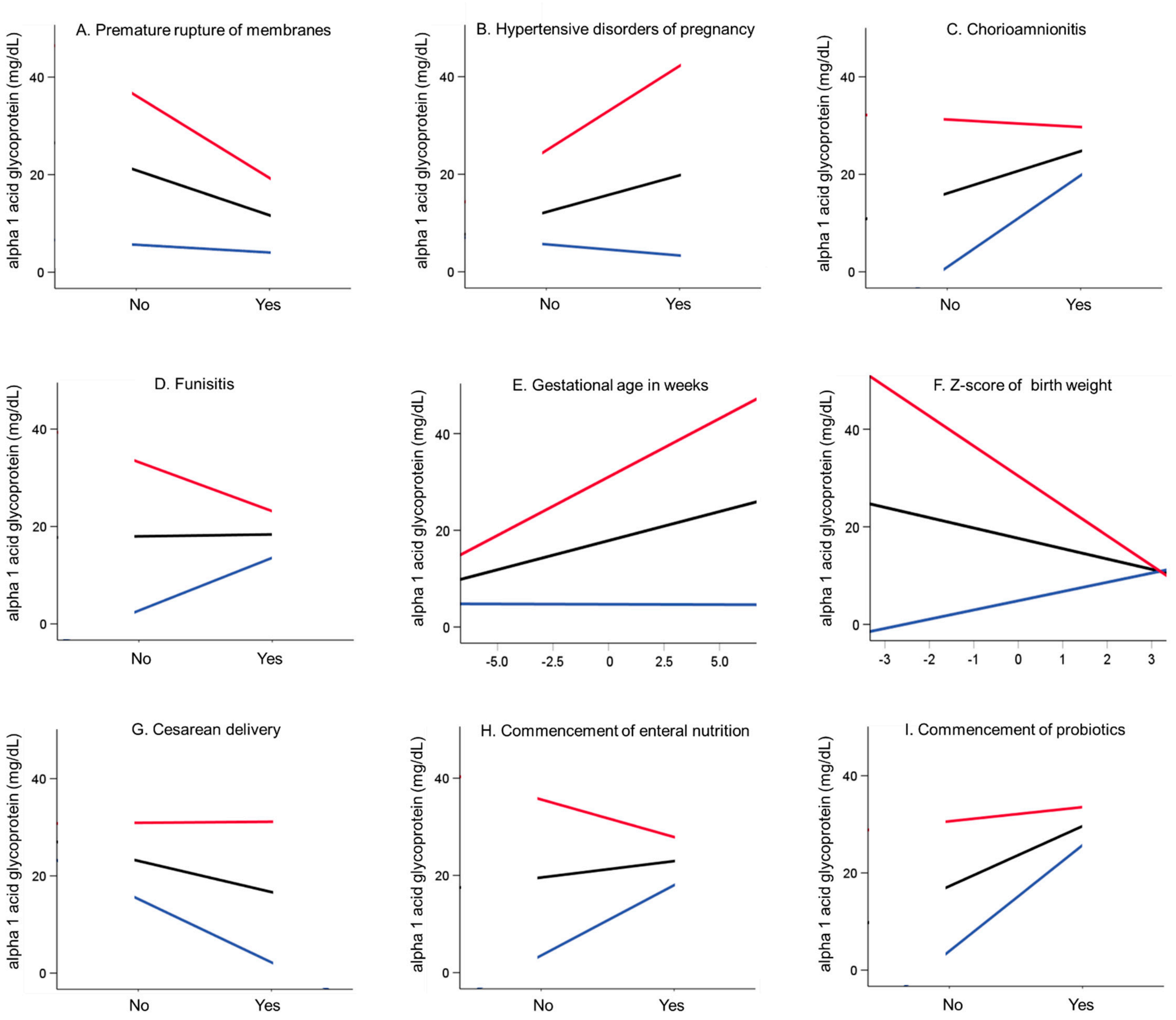

Regression lines between clinical variables and alpha 1 acid glycoprotein levels are shown for the early (blue line, one standard deviation below the mean), late (red line, one standard deviation above the mean), and mean (black line) postnatal ages using the simple slope analysis. Continuous variables (gestational age and z-scores of the body weight at birth) were mean-centered for convenience. See Table 4 for the confidence intervals and p-values.
